# Fenretinide-induced caspase-8 activation and apoptosis in an established model of metastatic neuroblastoma

**DOI:** 10.1186/1471-2407-9-97

**Published:** 2009-03-30

**Authors:** Gilda Raguénez, Annick Mühlethaler-Mottet, Roland Meier, Caroline Duros, Jean Bénard, Nicole Gross

**Affiliations:** 1 Centre National de Recherche Scientifique, Unité Mixte de Recherche 8126, Institut Fédératif de Recherche 54, Institut Gustave Roussy, Villejuif, France; 2 University Hospital CHUV, Pediatric Oncology Research, Department of Pediatrics, Lausanne, Switzerland; 3 Medical Biology and Pathology: Department, Institut Gustave Roussy, Villejuif, France; 4 Lawrence Berkeley National Lab, 1 Cyclotron Rd., MS-977-225A, University of California, Berkeley, CA 94720, USA

## Abstract

**Background:**

Resistance of high-risk metastatic neuroblastoma (HR-NB) to high dose chemotherapy (HD-CT) raises a major therapeutic challenge in pediatric oncology. Patients are treated by maintenance CT. For some patients, an adjuvant retinoid therapy is proposed, such as the synthetic retinoid fenretinide (4-HPR), an apoptotic inducer. Recent studies demonstrated that NB metastasis process is enhanced by the loss of caspase-8 involved in the Integrin-Mediated Death (IMD) process. As the role of caspase-8 appears to be critical in preventing metastasis, we aimed at studying the effect of 4-HPR on caspase-8 expression in metastatic neuroblasts.

**Methods:**

We used the human IGR-N-91 *MYCN*-amplified NB experimental model, able to disseminate *in vivo *from the primary nude mouse tumor xenograft (PTX) into myocardium (Myoc) and bone marrow (BM) of the animal. NB cell lines, *i.e.*, IGR-N-91 and SH-EP, were treated with various doses of Fenretinide (4-HPR), then cytotoxicity was analyzed by MTS proliferation assay, apoptosis by the propidium staining method, gene or protein expressions by RT-PCR and immunoblotting and caspases activity by colorimetric protease assays.

**Results:**

The IGR-N-91 parental cells do not express detectable caspase-8. However the PTX cells established from the primary tumor in the mouse, are caspase-8 positive. In contrast, metastatic BM and Myoc cells show a clear down-regulation of the caspase-8 expression. In parallel, the caspases -3, -9, -10, Bcl-2, or Bax expressions were unchanged. Our data show that in BM, compared to PTX cells, 4-HPR up-regulates caspase-8 expression that parallels a higher sensitivity to apoptotic cell death. Stable caspase-8-silenced SH-EP cells appear more resistant to 4-HPR-induced cell death compared to control SH-EP cells. Moreover, 4-HPR synergizes with drugs since apoptosis is restored in VP16- or TRAIL-resistant-BM cells. These results demonstrate that 4-HPR in up-regulating caspase-8 expression, restores and induces apoptotic cell death in metastatic neuroblasts through caspase-8 activation.

**Conclusion:**

This study provides basic clues for using fenretinide in clinical treatment of HR-NB patients. Moreover, since 4-HPR induces cell death in caspase-8 negative NB, it also challenges the concept of including 4-HPR in the induction of CT of these patients.

## Background

Neuroblastoma (NB), the second most common solid tumour in children, is an embryonic malignancy which originates from sympathetic neurons. Whereas localized tumors in young infants often spontaneously regress or mature in response to treatments, the outcome of high-risk NB, incurable in 60% of cases, remains poor and poses a major therapeutic challenge in pediatric oncology [1]. Apoptosis or programmed cell death is believed to represent an essential mechanism of drug-mediated toxicity [2]. Recent studies highlight the role of apoptosis in the metastatic process showing that defects in the apoptosis program contributed not only to malignancy or drug resistance but also to metastasis. Indeed, apoptosis is nowadays considered as a central mechanism in the development of metastases [3].

Apoptosis is an extremely well-organized process mediated by the caspases protein family. Upon activation by proteolytic cleavage, effector caspases cleave their substrates and inactivate proteins essential for survival, leading to the disintegration of cells [4]. The caspase-8 is a key enzyme at the top of the apoptotic cascade, both involved in the extrinsic or death receptors pathway and in the intrinsic mitochondrial pathway. Several studies have reported that the caspase-8 gene was frequently inactivated by hypermethylation in NB cell lines [5-7], alterations mainly described in aggressive and amplified *MYCN *high-stage tumors. However, no correlation has been established between caspase-8 expression and *MYCN *amplification or aggressive disease criteria [8]. Despite caspase-8 status is not predictive of aggressive NB, recent findings suggest that caspase-8 loss contributes to a metastatic phenotype, thus defining caspase-8 as a metastasis suppressor gene for NB [9]. The authors demonstrated that NB metastasis process is enhanced by the simultaneous loss of caspase-8 and of α3β1 integrin which subsequently impairs integrin-mediated death (IMD) to occur [10]. Therefore, caspase-8 appears as an attractive target to reduce the formation of NB metastases [11]. Among the numerous approaches manipulating caspase-8 levels, therapeutic strategies have used agents such as 5-Azacytidine [6], IFN-γ [12,13], IFN-γ associated with 5-Azacytidine [14], or the synthetic retinoid Fenretinide N-(4-hydroxyphenyl) retinamide/4-HPR [15]. Interestingly, 4-HPR has been reported to inhibit the invasion of several cancer cell types, including breast cancer, ovarian, prostate cancer and Kaposi's sarcoma, and NB [16]. Identified to induce apoptosis in various cell lines, the reagent 4-HPR-, -unlike retinoic acid (RA)-, -is more effective to induce apoptosis than differentiation in NB and proved a good efficacy against NB cell lines which are resistant to RA [17,18]. Moreover, 4-HPR is clinically well tolerated and used for clinical trials in neuroblastoma [19,20].

Our previous studies, performed in the experimental IGR-N-91 human *MYCN*-amplified model, reported the main involvement of P-gp/MDR in the chemoresistance of metastatic neuroblasts [21,22]. In this NB xenograft model, the IGR-N-91 neuroblasts have disseminated *in vivo *from the primary nude mouse tumor xenograft (PTX) into myocardium (Myoc) and bone marrow (BM) of the animal. Given the recent findings of i) potentiation of NB metastasis by loss of caspase-8 in a murine model and ii) halting NB metastasis by controlling IMD in the same model, we have investigated the expression of caspase-8 in metastatic neuroblasts of this experimental model. As we have observed a specific loss of caspase-8 expression in metastases, we therefore decided to restore caspase-8 activity with 4-HPR and to study its apoptotic-inducing effect in metastatic neuroblasts. Moreover, we have investigated the putative synergic effect of 4-HPR with cytotoxic drug- or TRAIL-mediated apoptosis in chemoresistant metastatic neuroblasts. Our results provide evidence that 4-HPR enhances cell death in caspase-8 deficient metastatic malignant neuroblasts and thus may be combined with toxic agents to an increased efficacy in the treatment of high-risk metastatic NB.

## Methods

### Cell culture and reagents

NB cell lines include the N-type NB IGR-N-91 cells and the S-type NB cells SH-EP. The IGR-N-91 cell line was established from a stage-4 NB infiltrated bone marrow [21]. Human IGR-N-91 NB cells were injected subcutaneously into nude mice: a primary tumor xenograft cell line (PTX), blood cells (BC), myocardium (Myoc) and bone marrow (BM) sub-lines were obtained from mechanically dissociated tumor samples and were grown as previously described [21,22]. Each cell line was cultured in Dulbecco's Modified Eagle Medium (DMEM) supplemented with 2 mM L-glutamine, 10 μg/ml gentamycin and 10% fetal calf serum, at 37°C in a 5% CO2 humidified atmosphere.

4-HPR (from Sigma-Aldrich, Germany) was dissolved in ETOH. Throughout this study, we used 1–10 μM of 4-HPR for the N-type NB cells and 1–20 μM for the S-type NB cells. The concentrations were sufficient to induce cell death in NB cells as determined previously [23,24]. Cells were treated with 20–40 μM etoposide (VP16) (Sigma, St Louis, MO), both dissolved in DMSO, for 24 or 48 hours. An equal volume of vehicle control (DMSO) was used to treat control cells. Cells were incubated with indicated amount of soluble recombinant TRAIL (a gift from J. Tschopp) and cross-linking anti-Flag Ab M2 (Sigma) with a constant ratio of 1/5 of TRAIL/M2 as previously described [25]. Cells were pretreated with caspase inhibitors zIETD-fmk, zDEVD-fmk, zVDAVD-fmk and zLEHD-fmk (50 μM, R&D systems SA, Switzerland) before drug treatment.

### Tumor growth

Athymic locally bred Swiss nude mice were engrafted with the IGR-N-91 cell line, in accordance to the European Community guidelines (authorization #86/609/CEE). Mice (n = 4) were anaesthetized using ketamine 50 mg/kg and xylazine 35 mg/kg intraperitoneally (I.P.). Heterotopic tumors were obtained by subcutaneous injection in the back of the animal of 1 × 10^6 ^NB cells suspended in 200 ml of Matrigel^® ^(BD Biosciences, San Diego, CA) using a 22 G needle connected to a 1-ml syringe. After recovery, the animals were kept in a germ-free protected area, with food ad libitum. Mice were sacrificed after 2 months. Tumors were resected and dissociated as described previously [26]. Proteins were isolated using lysis buffer as described above.

### Retroviral infection experiments

IGR-N-91 cells were infected with a biscistronic retroviral vector encoding for the GFP and the caspase-8a genes (IGR-N91-C8) or with the GFP control vector (IGR-N91-M) as described previously [25]. One clone was respectively chosen among the GFP positive IGR-N91-M clones and among the IGR-N91-C8 clones, then assessed to control the expression level of caspase-8 by immunoblotting. Stable downregulation of caspase-8 was achieved by RNA interference using short hairpin RNAs [27]. Different splice variants of caspases-8 mRNA were identified leading to the production of diverse C8 isoforms [28]. To efficiently silence all caspase isoforms, the shRNAs have been localized in common exons and correspond to the sequence 5'-AAGTTCCTGAGCCTGGACTAC-3' as proposed previously [29]. The sequence of oligonucleotides encoding the control random shRNA (shRNAC) is 5'-CGATTGCGATCGTCAATTCCC-3' [30]. The sequences were checked by sequencing a PCR-amplified region containing the oligonucleotide and shRNAs were prepared and used to infect the SH-EP cell line as already described [30].

### Cell viability and apoptosis assays

Cell viability was measured using the MTS/PMS cell proliferation kit from Promega (France) according the manufacturer's instructions. 10^5 ^cells were seeded 24 hours into a 96-well plate containing 100 μl of medium, then incubated with 4-HRP or drugs for 24 or 48 hours. Assays were performed in quadruplicates. Percentage of cell viability/cell death was determined as compared to untreated controls. Apoptosis was evaluated using the standard PI-staining protocol. Cells were trypsinized and neutralized in complete medium, washed twice with 3 ml PBS. Cell pellets were resuspended in 1 ml of PBS on ice then fixed in 3 ml of 100% ethanol. After fixation, pellets were kept at -20°C. Cells were stained in PI solution (50 μg/ml PI, 100 μg/ml RNAseA in PBS) for 30 min at room temperature. The stained cells were analyzed using a FACScan flow cytometer (Becton Dickinson, France SAS).

### Western Blotting

Western blots were performed on cultured cells homogenized in RIPA buffer (50 mM Tris-HCl, pH 7.5, 150 mM NaCl, 1% NP40, 0.5% DOC, 0.1% SDS) on ice for 15 minutes. Protein extracts (50 μg/lane) were loaded on 10–12% SDS-PAGE, transferred on nitrocellulose membranes. Immunoblots were saturated with 5% skim milk, 0.1% Tween-20 in TBS and revealed using mouse monoclonal antibodies or rabbit polyclonal antibodies to detect Bcl-2 (C124, Dako France), Bax (BD Pharmingen Inc., San Diego, USA), procaspase-3 (BD Pharmingen), procaspase-8 (MBL International, USA), procaspase-9 (Cell Signaling Technology, Inc., USA), procaspase-10 (MBL International), β-actin (Sigma). Bound antibodies were revealed by incubation with either goat anti-mouse IgG (Jackson ImmunoResearch Laboratories, Inc., Europe) or goat anti-rabbit IgG (Nordic Immunological Laboratories, Netherlands), and subsequently detected using the Lumilight Western Blotting substrate (Roche Diagnostics, Switzerland,) according to the manufacturer's instructions.

### Caspases activities

Caspases-3, -8, and -9 protease activities were measured using the caspase colorimetric protease assay kits from MBL Co., Ltd, USA as previously described [25]. Cytosolic lysates were prepared 24 or 48 hrs after 4-HPR treatment according to manufacturer's instructions. 100 μg protein extracts were incubated with 200 μM of DEVD-pNA, IETD-pNA, Ac-LEDH-pNA colorimetric substrates for 3 hrs at 37C. Cell lysates were incubated with 10 μM of caspase inhibitor (zDEVD-fmk, zIETD-fmk, zLEDH-fmk) for 30 min before addition of the respective caspase substrates for control experiments. Hydrolyzed pNA was detected using a microtiter plate reader at 405 nm. Background absorbance from cell lysates and buffers were subtracted from the absorbance of stimulated and unstimulated samples before calculation of relative caspases activities.

### RT-PCR experiments

Total RNA was isolated using RNAble reagent (Eurobio, France). Reverse transcription was performed from 1 μg total RNA using Superscript II RNase H-Reverse Transcriptase (Gibco BRL). PCR reactions were performed on 1 ng of cDNA using the Platinum Taq polymerase (Invitrogen Corporation, Switzerland) in a PTC100 thermocycler (MJ Research, Inc., USA). The sequences of primers used were as follows: for caspase-8 (forward: 5'-TCTGGAGCATCTGCTGTCTG-3'; reverse: 5'-CCTGCCTGGTGTCTGAAGTT-5') and for GAPDH (forward: 5'-CTGCACCAACTGCTTAG-3', reverse: 5'-AGGTCCACCACTGACACGTT-3'). The PCR products were analyzed by 1.5% agarose gel electrophoresis. The size of PCR fragment is 356 bp.

### Statistical Analysis

Results are shown as the mean ± SD of at least three experiments each. Data were analyzed using the Student's *t *test with *P *values of <0.05 (*) or <0.01 (**) considered to represent significance.

## Results

### Loss of caspase-8 expression in metastatic neuroblasts

**Figure 1:**
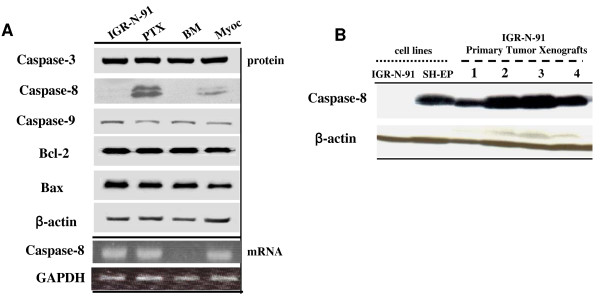
**Caspase-8 expression in the IGR-N-91 experimental metastatic model. A) **Western Blot analyses of caspases-3, -8, -9, Bcl-2 and Bax expressions in cell lines of the IGR-N-91 experimental model. Semi-quantitative RT-PCR analyses of caspase-8 mRNA expressions and GAPDH as internal control. PTX: Primary Tumor Xenograft, BM: Bone Marrow, Myoc: Myocardium. **B) **Whole extracts from the IGR-N-91 and SH-EP cell lines, and four IGR-N-91 primary tumor xenografts were analyzed by immunoblotting for caspase-8 expression. β-actin was used as loading control.

We have first analyzed caspase-8 expression level in PTX, BM and Myoc cell lines of the IGR-N-91 experimental model (Figure 1A). We observed that the IGR-N-91 cell line is caspase-8-negative. Surprisingly, caspase-8 expression is restored in PTX compared to parental IGR-N-91 neuroblasts, then downregulated in BM or Myoc metastatic neuroblasts. RNA analysis revealed caspase-8 mRNA transcripts in parental IGR-N-91 cells as well as in Myoc neuroblasts while totally absent in BM cells (Figure 1A). In contrast, no change was observed in the expression levels of caspases-3, -9 as well as Bcl-2 and Bax. Therefore, we examined by immunoblotting caspase-8 expression level in four primary tumors xenografts of mouse engrafted with IGR-N-91 neuroblasts (Figure 1B). The SH-EP cell line was used as a caspase-8-positive control cell line. As for PTX neuroblasts, caspase-8 expression is fully restored in the four primary tumors.

### 4-HPR induced re-expression of caspase-8 and apoptotic cell death in BM metastatic neuroblasts

**Figure 2:**
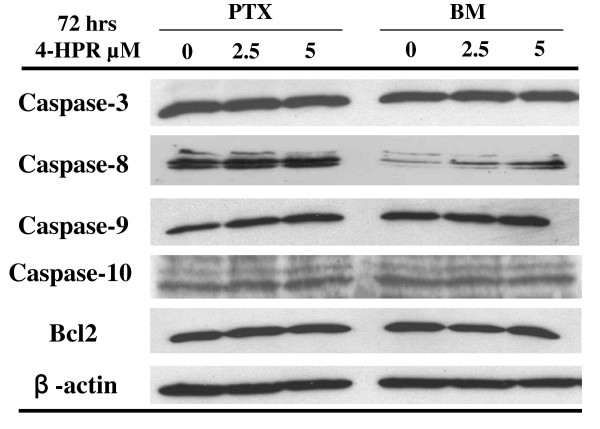
**4-HPR induces caspase-8 expression in BM metastatic neuroblasts**. Western Blotting of caspases-3, -8, -9, -10 and Bcl-2 expressions in PTX and BM neuroblasts treated with 2.5 or 5 μM 4-HPR.

**Figure 3:**
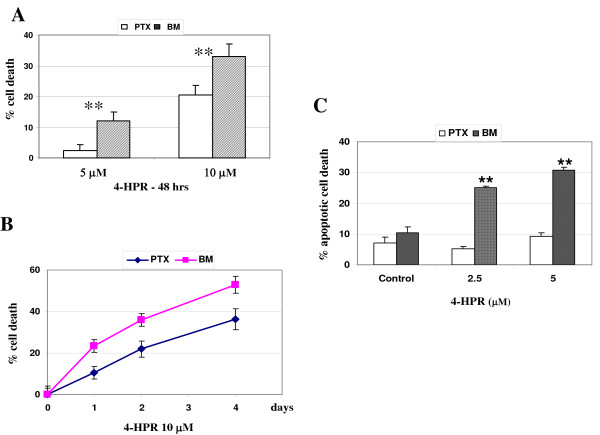
**4-HPR induces apoptosis in BM metastatic neuroblasts**. **A-B) **Dose-response and time-course analysis of 4-HPR-induced cell death in PTX and BM neuroblasts. Cell viabilities assessed by MTS analysis in PTX and BM neuroblasts treated with 5 or 10 μM 4-HPR for 48 hours **(A) **or with 10 μM 4-HPR for 1 to 4 days **(B)**. The values reported are mean ± SD of three independent experiments. ** *P *< 0.01 indicates difference between PTX and BM neuroblasts. **C) **The increase of apoptotic cell death is correlated with caspase-8 re-expression in BM metastatic cells. Sub-G1 apoptotic cells were detected by the propidium staining method after stimulation for 72 hours with 2.5 or 5 μM 4-HPR. Mean values ± SD of 3 independent experiments are shown.** *P*< 0.01 indicates significant difference between PTX/BM cells and respective untreated cells.

As the role of caspase-8 appears critical in preventing metastasis, we aimed at studying the effect of the retinoid 4-HPR on caspase-8 expression. Figure 2 shows a specific up-regulation of the caspase-8 protein level in BM metastatic neuroblasts treated with 2.5 or 5 μM 4-HPR for 72 hours, while no change was observed in PTX cells. No change was noted in the levels of caspase-3, -9, -10 and Bcl-2 expressions in PTX or BM neuroblasts. Additionally, MTS analysis was performed in PTX and BM neuroblasts treated with 5 or 10 μM 4-HPR for 48 hours and showed that PTX cells are more resistant to cell death than BM cells (Figure 3A). Indeed, 4-HPR-induced cell death is dose- and time-dependent (Figure 3A, B). As 4-HPR has been previously described to induce apoptotic cell death in neuroblastoma cell lines [23], we have compared the extent of 4-HPR-mediated apoptosis in PTX and BM neuroblasts. FACS analysis shows that apoptosis was significantly increased in BM neuroblasts, whereas no apoptosis occurred in PTX cells (Figure 3C). Thus, the increase of the caspase-8 expression appears correlated with a higher sensitivity of apoptotic cell death in BM compared with PTX neuroblasts.

### Caspase-8 activation by 4-HPR in BM metastatic neuroblasts

**Figure 4:**
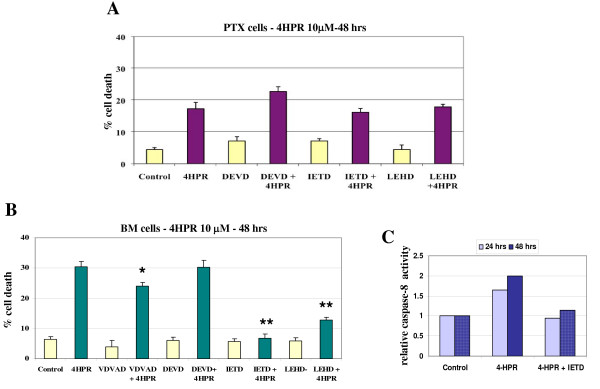
**Caspase-8 activation in BM metastatic neuroblasts**. **A-B) **Cell viabilities assessed by MTS analysis in PTX cells **(A) **and in BM cells **(B) **pretreated with 50 μM of caspase inhibitor (zVDAVD-fmk, zIETD-fmk, zLEHD-fmk, zDEVD-fmk) for 1 hr, then treated with 10 μM 4HPR for 48 hrs. Data points show mean ± SD of 3 independent experiments. *P < 0.05, **P < 0.01 compared with control cells treated with 4-HPR only. **C) **Caspase-8 activity was measured using lysates from BM cells treated with 10 μM 4-HPR for 24 or 48 hrs. Activity was normalized to the activity in the untreated cells (control), and blocked by addition of 10 μM caspase-8 inhibitor (zIETD-fmk).

In order to analyze the involvement of caspase-8 in cell-death execution, we conducted studies using specific inhibitors. The 4HPR-induced cell death is not significantly blocked in PTX cells by zDEVD-fmk (a caspase-3 inhibitor), zIETD-fmk (a caspase-8 inhibitor), or zLEHD-fmk (a caspase-9 inhibitor) (Figure 4A). In contrast, we observed that 4HPR-induced cell death can be blocked by zIETD-fmk (*P *< 0.01), more partially by zLEDH-fmk (P < 0.01) and slightly by zVDAVD-fmk (a caspase-2 inhibitor) (*P *< 0.05) in BM cells. However, no inhibition of cell death was noted with the caspase-3 inhibitor zDEVD-fmk (Figure 4B). The caspases-3, -8 and -9 activities were measured in BM metastatic cells incubated with 10 μM 4-HPR for 24 or 48 hours. A slight increase of caspase-8 activity is noted within 24 hrs, appears more sustained after 48 hours, and is reduced by the caspase-8 inhibitor zIETD-fmk (Figure 4C). However, no significant variation of caspases-3 or -9 activities was evidenced in these conditions (data not shown).

### Caspase-8 negative NB cell lines are resistant to 4-HPR-induced cell death

**Figure 5:**
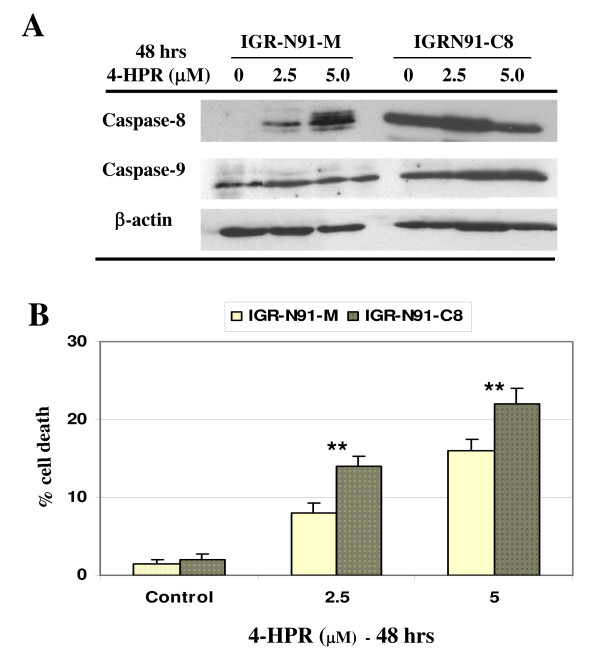
**IGR-N91 neuroblasts sensitivity to 4-HPR-induced cell death**. **A) **The IGR-N91-M control cells and IGR-N91-C8 clone 19 cells were treated with 2.5 or 5 μM 4HPR for 48 hrs. Cell lysates were analyzed by IB for caspase-8, -9 and β-actin as loading control. **B) **Cell death was determined by the MTS assay and the data represent the mean values of two representative experiments ± SD. **P < 0.01 indicates difference between 4HPR-treated cells and EtOH-treated cells.

To corroborate the specific contribution of caspase-8 in 4-HPR-induced apoptosis, we have used the caspase-8-deficient NB cell line (IGR-N91-M) and the stably restored caspase-8 cell line (IGR-N91-C8) previously described [25]. We observed that the treatment of 2.5 or 5 μM 4-HPR strongly up-regulated caspase-8 expression in the IGR-N91-M neuroblasts, while no change was observed in the IGR-N91-C8 cells (Figure 5A). In parallel, significant cell death may be noted both in 4HPR-treated IGR91-M and IGR91-C8 neuroblasts (Figure 5B). Thus, the increase of caspase-8 expression may be correlated with the higher cell death sensitivity noted in the 4HPR-incubated IGR91-M cells compared with control.

**Figure 6:**
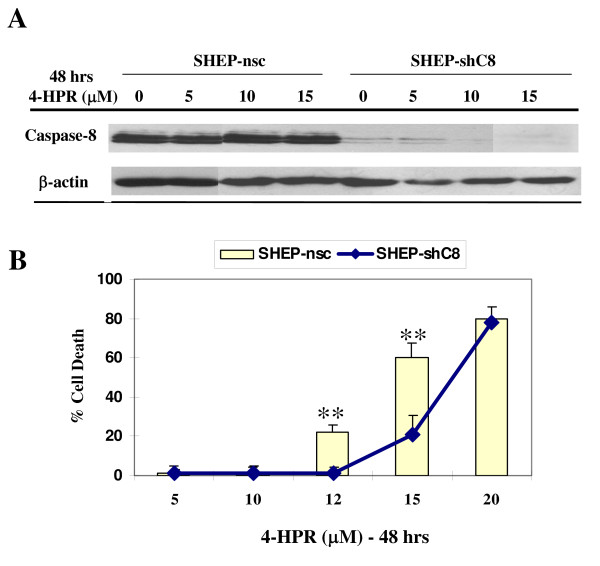
**Caspase 8-negative SH-EP neuroblasts are resistant to 4-HPR-induced cell death**. **A) **SH-EP cells infected with shRNA targeting caspase-8 (shC8) or nonsilencing control (nsc) were treated with 5, 10 or 15 μM 4-HPR for 48 hrs. Cell lysates were analyzed by western blotting for caspase-8 and β-actin used as loading control. **B) **Cell death was measured by MTS cell proliferation assays in the SH-EP cell lines. Mean values of three independent experiments ± SD are shown. ** *P*< 0.01 indicates difference between SHEP-nsc and SHEP-shC8 cells.

To extend the observation that caspase-8 activation plays a major role in 4-HPR-induced cell death, we examined the response of SH-EP cell line to 4-HPR, after silencing of caspase-8 expression. SH-EP cells were stably transfected with shRNAs against caspase-8 (SHEP-shC8) and non-silencing control (SHEP-nsc). As expected, western blot analysis shows that caspase-8 is silenced in SHEP-shC8 (Figure 6A). The two cell lines were then treated with increasing doses of 5, 10 or 15 μM 4-HPR for 48 hours. The 4HPR treatment did not change caspase-8 expression neither in SHEP-nsc or SHEP-shC8 cells (Figure 4B). Cell death was measured using the MTS test (Figure 6B). Interestingly, the SHEP-shC8 cells appear significantly more resistant compared to SHEP-ncs cells, thus confirming that 4-HPR-induced cell death requires caspase-8 expression and activation.

### 4-HPR enhances chemotherapeutic drug- or TRAIL-mediated toxicity in metastatic neuroblasts

**Figure 7:**
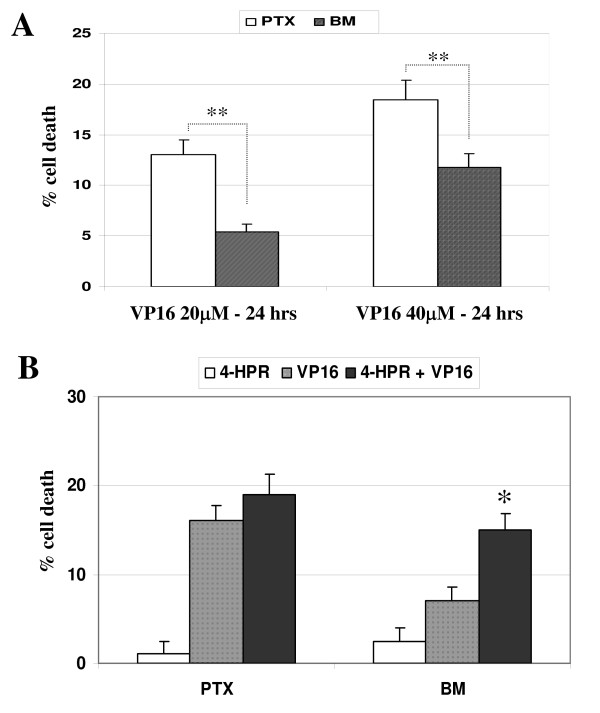
**4-HPR enhances VP16-mediated cytotoxicity in BM metastatic neuroblasts**. **A) **PTX and BM cells were treated with 20 or 40 μM etoposide/VP16 for 24 hrs. Cell death was measured as previously described and the data are expressed as a percentage of control cells. Mean ± SD of three independent experiments. **P < 0.01 indicates significant difference between PTX and BM cells. **B) **PTX and BM cells were incubated with 2.5 μM 4-HPR for 96 hrs, then with 20 μM VP16 for 24 hrs. Data points show mean ± SD of a representative experiment. The (*) indicates that synergism was demonstrated for VP16 and 4HPR.

**Figure 8:**
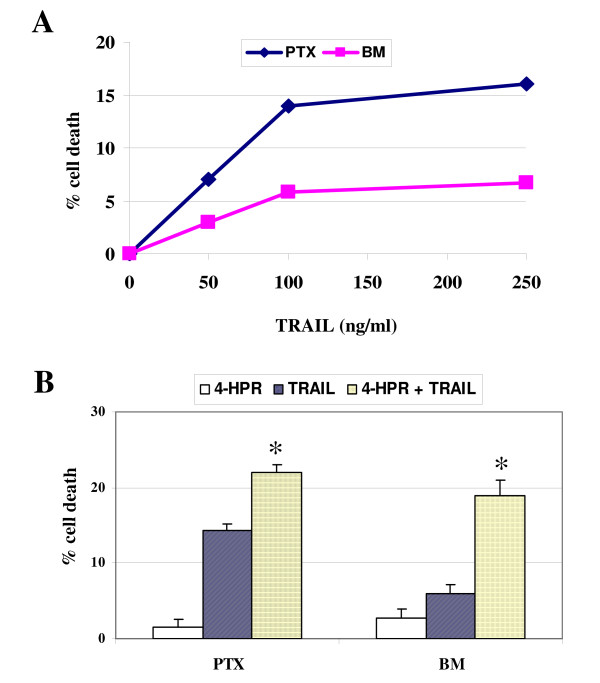
**4-HPR enhances TRAIL-mediated cytotoxicity in BM metastatic neuroblasts**. **A) **PTX and BM neuroblasts were incubated with increasing concentrations of TRAIL for 48 hrs, as indicated. BM cells are more resistant than PTX cells to TRAIL-mediated toxicity. Data points show mean ± SD of three independent experiments. **B) **PTX and BM cells were incubated with or without 4-HPR (2.5 μM) for 96 h, and TRAIL was added to a final concentration of 250 ng/ml for the final 48 h in culture. Mean ± SD of three independent experiments. The (*) indicates that synergism was demonstrated at these concentrations of TRAIL and 4HPR.

Next, we have investigated if 4-HPR could enhance drug-mediated apoptosis in BM metastatic neuroblasts, which, contrary to what occurs with 4-HPR, are more resistant than PTX neuroblasts to toxicity by drugs. Indeed, we had previously shown that BM metastatic neuroblasts are resistant to etoposide/VP16 toxicity [22]. Figure 7A confirms that BM are more resistant than PTX cells to drug toxicity after incubation of cells with 20 or 40 μM VP16 for 24 hours. PTX and BM neuroblasts were pretreated with 2.5 μM 4-HPR for 96 hours and data show that 4-HPR enhances VP16-mediated toxicity in BM cells (Figure 7B). These data are in accordance with a previous study showing a synergistic apoptotic response of 4-HPR with chemotherapeutic agents, including cisplatin, carboplatin or etoposide in NB [31]. Caspase-8-positive NB cells have been previously demonstrated to be weakly sensitive to TRAIL [25]. Moreover, studies reported silencing of caspase-8 expression in malignant IGR-N-91 cells as a possible mechanism of resistance to TRAIL-induced apoptosis. Indeed, sensitivity to TRAIL was fully restored in the caspase-8-complemented NB cell line [32]. Here we analyzed the cell death response of BM cells to TRAIL-mediated toxicity compared with PTX cells (Figure 8A). The caspase-8-silenced BM neuroblasts appear more resistant than PTX neuroblasts to TRAIL-toxicity. Moreover, we observed a more important enhancement of TRAIL-mediated toxicity in BM cells than in PTX after incubation with 2.5 μM 4-HPR for 96 hours (Figure 8B). These data corroborate previous studies showing that 4-HPR enhances TRAIL-mediated apoptosis in ovarian or colon cancer cells [33,34].

## Discussion

Numerous studies have reported that the loss of caspase-8 expression occurs in medulloblastoma [35], colon carcinoma [36], more frequently in small cell lung carcinoma, glioma and malignant neuroblastoma [37,38,6]. In high-risk neuroblastoma, resistance to apoptosis may involve several defects of the apoptotic pathway, such as over-expression of the anti-apoptotic proteins Bcl-2, Bcl-x_L_, and survivin [39], but also deregulation of the death receptor pathway with the inactivation of *caspase-8 *gene expression mostly by gene silencing [5,6,40].

The present study shows that the downregulation of caspase-8 plays a role in resistance of metastatic neuroblastoma to apoptosis, which could be reversed by 4-HPR treatment. Since 4-HPR upregulates caspase-8 expression, apoptotic resistance induced by 4-HPR appears improbable. We have investigated the expression of caspase-8 in the IGR-N-91 NB experimental model which offers the opportunity to compare NB cell lines established from a primary tumor and matched metastatic neuroblasts [21,22]. In this model, the IGR-N-91 parental cell line is negative for caspase-8 protein despite normal level of mRNA. This suggests the occurrence of a post-transcriptional mechanism to shut off caspase-8 protein expression in parental IGR-N-91 cells. We observed that caspase-8 protein is re-expressed in the primary tumor cell line (PTX) and both caspases-8 mRNA and protein expressions are lost in metastatic neuroblasts (BM and Myoc). As known and previously described [6], the role of methylation in the caspase-8 silencing process is supported by the up-regulation of caspase-8 observed in NB cell lines treated with demethylating agents such as 5-azacytidine. Thus, changes in methylation and/or chromatin structure might explain the caspase-8 silencing in NB metastatic cells, and inversely the re-expression of caspase-8 in tumors with the contribution of others mechanisms such as interactions with the cellular microenvironment. Moreover, caspase-8 seems to not impact primary tumor growth as the cells interact with each other and with the remodeling tumor ECM, caspase-8 being able to regulate tumor survival during tissue invasion [41]. The loss of caspase-8 is specific since the expression of other caspases or apoptotic partners was unchanged. Interestingly, our data are consistent with recent findings which first, correlate the decrease of apoptosis induction due to the loss of caspase-8 with the increase of metastatic potential in NB [9], and secondly, with the loss of caspase-8 or integrins allowing malignant disseminated cells to acquire a greater potential to survive [10].

As the role of caspase-8 appears to be crucial in preventing metastasis, we therefore decided to study the effect of the synthetic retinoid fenretinide/4-HPR on caspase-8 expression in metastatic neuroblasts. *In vitro *studies in NB have shown that 4-HPR induced apoptosis through RAR-dependent or -independent pathways [23,24]. The activation of c-Jun N-terminal kinase as well as the activation of mitochondrial pathway via generation of reactive oxygen species (ROS) or the induction of increased ceramide production have been implicated in 4HPR-mediated apoptosis [42,43]. Here, we report that 4-HPR induces apoptosis both in PTX and BM malignant neuroblasts. Interestingly, 4-HPR up-regulates caspase-8 expression in caspase-8 negative IGR-N91 cells, *i.e*, BM neuroblasts or retrovirally-transduced IGR-N91-control cells. Moreover, this up-regulation is correlated with a significant increase of apoptotic cell death in BM neuroblasts. Data confirm that BM metastatic neuroblasts are more sensitive to 4-HPR-induced cell death than PTX cells and that caspase-8 activation occurs in BM neuroblasts treated by 4-HPR. This higher sensitivity of BM cells to 4-HPR may be explained by two mechanisms: first, the involvement of IMD impairment in the apoptotic resistance of metastatic BM cells. Interestingly, we have observed a decrease of the expression levels of the α3 and β1 integrins in BM or Myoc metastatic cells compared with parental IGR or PTX cells (data not shown). As 4-HPR induces an increase of caspase-8 expression, it would be informative to accurately investigate if 4-HPR is able to restore IMD, *i.e *not only caspase-8 but also α3β1 integrin expressions. Thus, the higher sensitivity of BM cells to 4-HPR might be imputed to a synergistic effect of both intrinsic 4-HPR toxicity and restored IMD; secondly, studies using specific inhibitors show that 4HPR-induced cell death is almost completely inhibited by the addition of zIETD-fmk, a caspase-8 inhibitor and more partially with zLEHD-fmk, a caspase-9 inhibitor. In contrast, these inhibitors did not protect PTX cells for 4-HPR toxicity, suggesting that cell death induced by 4-HPR occurred by distinct pathways in PTX cells compared to BM cells.

Previous findings reported that 4-HPR stimulates the generation of intracellular free radicals which has a causative role in the induction of apoptosis and that caspase-3 activation is required in this oxidative stress-mediated apoptosis [24,44]. However, in our study, the caspase-3 inhibitor does not inhibit 4-HPR-induced apoptosis in BM neuroblasts, suggesting that other pathways may be involved. If 4-HPR-induced BM cell death is partially inhibited by the caspase-2 inhibitor, further studies are clearly needed to elucidate the mechanism of 4HPR/caspases interactions involved in this experimental model. Nevertheless, the crucial role of caspase-8 in 4-HPR mediated death in BM cells is confirmed by data performed with the transfected and silenced pair of IGR-N91-M/C8 and SH-EP-nsc/shC8 cells. As observed in the PTX/BM pair of cells, caspase-8 expression was increased by 4-HPR in the caspase-8 deficient cells IGR-N91-M cells, whereas in SH-EP-shC8, caspase-8 over-expression could not be detected because C8 mRNA was degraded by the shRNA. Interestingly, the stable restoration of caspase-8 expression in IGR-N91-C8 cells increased their sensitivity to 4-HPR compared to parental IGR-N91-M cells, while the reversed experiment, performed by down-regulating caspase-8 expression in SH-EP-shC8, increased their resistance to 4-HPR compared to parental SH-EP-nsc.

Similar caspase-8 activation has been described in 4-HPR-induced cell lines such as ovarian cancer cell lines or Fas-defective hepatoma cells [33,45]. More recently, different retinoic acid derivatives, including 4-HPR, were demonstrated to increase caspase-8 transcription via induction of phosphorylated CREB in NB [46]. Moreover, the authors demonstrate that the binding of this transcription factor to sequences within the caspase-8 gene sensitizes cells to TNFα or drug-induced apoptosis [46]. Our study corroborates that caspase-8 appears as a key mediator of the synergistic effect observed between RA derivatives/4HPR and drugs/etoposide or TRAIL. Indeed, 4-HPR represents a potential clinical candidate to enhance the effect of chemotherapy in NB [31]. However, this effect was imputed to the generation of free radicals by 4-HPR, considered as the key property of this retinoid leading to synergistic response with chemotherapy [31]. Resistance to TRAIL-mediated cell death has been attributed to deregulation of a number of signalling molecules [47], including inactivation of caspase-8 [5-7]. Indeed, restored caspase-8 expression results in an increase of sensitivity of NB cells in TRAIL-induced apoptosis [25]. Here, we report that the caspase-8-negative BM neuroblasts are more resistant to TRAIL-mediated cell death than PTX cells. Moreover, a more important enhancement of TRAIL-induced cell death is observed in BM neuroblasts pre-treated with 4HPR. Recent studies performed in colon cancer cell lines described such a synergistic effect of the combination of TRAIL with 4-HPR leading to the activation of multiple caspases [34].

Both previous reports and our study propose the restoration of caspase-8 function as essential to sensitize resistant NB to apoptosis. However, NB malignant cells are characterized by a great heterogeneity and show various resistance or sensitivity to TRAIL or drug toxicities. Thus, studies have always to be performed to develop new and various combination regimens for high-risk neuroblastoma. Indeed, previous findings reported the caspase-8 induction by IFN-γ in selected NB cells [12], able to sensitize NB to TRAIL apoptosis [48]. More recently, the association of the demethylating agent 5-Aza-2'-deoxycytidine (5-dAzaC) with IFN-γ was described to cooperate using lower individual drug doses in order to increase the therapeutic efficacy and decrease the toxicity of TRAIL-apoptosis in NB [14].

## Conclusion

Our study confirms that 4-HPR/Fenretinide is a very interesting therapeutic drug, reported to exert a benefit effect in the treatment of prostate cancer metastasis or to suppress the invasiveness of bone metastatic breast cancer cells [49,50]. Of note, fenretinide is currently studied for a phase II trial in HR-NB patients following a successful phase I trial and recent pharmacokinetics studies conducted in NB patients indicated that a continuous oral administration of 4-HPR is preferred to maintain a relevant and efficient plasma concentration of 0.7 -10 μM range [19, 51)]. Moreover, active metabolites of 4-HPR such as 4-oxo-4-HPR are currently under investigation and may provide a promising therapy for HR-NB [51].

## Abbreviations

NB: neuroblastoma; CT: chemotherapy; TRAIL: Tumour Necrosis Factor-related apoptosis-inducing ligand; TNFα: Tumor Necrosis Factor alpha; 4-HPR: *N*-(4-hydroxyphenyl) retinamide; MTS: 83-(4,5-dimethylthiazol-2-yl)-5-(3-carboxymethoxyphenyl)-2-(4-sulfophenyl)-2H-tetrazolium).

## Competing interests

The authors declare that they have no competing interests.

## Authors' contributions

GR performed major experimental work, conceived of the study in its design and coordination, and drafted the manuscript. AM performed and characterized the stable IGR-N91-M and -C8 and SHEP-nsc and -shC8 cell lines. CD performed FACS analysis and immunoblotting experiments. RM carried out the experiments in mice. AM, JB and NG were involved in the overall design of the study and helped to draft the manuscript. All authors read and approved the final manuscript.

## Pre-publication history

The pre-publication history for this paper can be accessed here:

http://www.biomedcentral.com/1471-2407/9/97/prepub
